# Gambling-Related Cognitive Distortions Among Adolescents: A Gender-Based Analysis

**DOI:** 10.1007/s10899-026-10498-z

**Published:** 2026-04-01

**Authors:** Gema Aonso-Diego, Aida María Iglesias-Faba, Ana Estévez

**Affiliations:** Department of Psychology, Faculty of Health Sciences, University of Deusto, Avda. de las Universidades, 24, Bilbao, 48007 Spain

**Keywords:** Gambling, Cognitive distortions, Illusion of control, Online gambling, Modality, Adolescents

## Abstract

Cognitive distortions represent one of the most extensively studied cognitive factors in relation to gambling behavior. This study examined gambling-related cognitive distortions across gambling severity levels, gambling modalities, and the type of gambling, while also exploring the mediating role of cognitive distortions in the relationship between gambling modality, gambling type, and gambling severity. All analyses were conducted separately for men and women to identify gender differences in the adolescent population. A total of 209 past-year gamblers enrolled in secondary school, aged between 14 and 18 years (Mage = 15.72; SD = 1.05; 69.1% boys) participated in the study. Gambling severity was assessed using the adolescent-adapted version of the South Oaks Gambling Screen (SOGS-RA) and cognitive distortions were measured through the Gambling Belief Questionnaire (GBQ). Cognitive distortions increased with gambling severity (p = .003) and were higher among online gamblers (p = .003). Gender-stratified analyses indicated that women differed by gambling modality, with higher distortions in online gamblers (p = .009), whereas men differed by severity level, with greater distortions in those with problematic gambling (p = .003). Model indicated that cognitive distortions mediated the association between online gambling and severity, with total mediation in women (β = 0.651, p = .035) and partial mediation in men (β = 0.399, p = .028). These findings underscore the importance of incorporating gender-sensitive and context-specific components into adolescent gambling prevention and intervention programs.

## Introduction

Rooted in the cognitive model of gambling behavior, cognitive distortions are considered a central risk factor in the etiology and maintenance of gambling problems (Goodie et al., 2019). These distortions refer to thinking errors or irrational beliefs about chance, skill, and control, which lead to misinterpretations of gambling outcomes and motivate individuals to initiate or persist in gambling despite repeated losses. Several of these cognitive distortions stem from the heuristics described by Tversky and Kahneman (1974), who proposed that people rely on cognitive shortcuts to make judgments under uncertainty. While heuristics often facilitate efficient decision-making, they can also lead to systematic errors or biases. In the gambling context, many distortions arise from the representativeness heuristic (e.g., the gambler’s fallacy, overconfidence, or trends in number picking), and the availability heuristic (e.g., illusory correlations, the availability of others’ wins, or inherent memory bias), as individuals misinterpret random events or recall past outcomes selectively. Other distortions, such as the illusion of control or near-miss effects, have been widely documented but are not directly derived from these heuristic processes (see Fortune & Goodie, 2012 for a review).

Empirical research has consistently demonstrated a strong association between cognitive distortions and gambling behavior (Fortune & Goodie, 2012; Goodie et al., 2019). Cognitive distortions have been linked to several gambling-related variables, including greater gambling severity (Bonnaire et al., 2022; Calado et al., 2017; Ciccarelli et al., 2017, 2017; Cosenza et al., 2019; Esparza-Reig et al., 2023; Mancone et al., 2025; Orlowski et al., 2020; Thurm et al., 2022), higher gambling frequency (Donati et al., 2019; Mancone et al., 2025; Nigro et al., 2021), and an earlier age of onset (Edgerton et al., 2015). Most studies have indicated that individuals with problematic gambling exhibit higher levels of cognitive distortions compared to those with non-problematic gambling (Bonnaire et al., 2022; Ciccarelli et al., 2017; Donati et al., 2018; Labrador et al., 2020; Mancone et al., 2025; Mathieu et al., 2020; Matsushita et al., 2025). Notably, this finding has been reported both in adolescent (Calado et al., 2017; Ciccarelli et al., 2021; Cosenza et al., 2019) and adult populations (Bonnaire et al., 2022; Ciccarelli et al., 2017; Labrador et al., 2020; Nigro et al., 2021); in both cross-sectional (Bonnaire et al., 2022; Ciccarelli et al., 2017; Cosenza et al., 2019; Donati et al., 2018) and longitudinal designs (Cowie et al., 2017; Koga et al., 2025; Philander & Gainsbury, 2023); as well as in clinical (Koga et al., 2025; Mallorquí-Bagué et al., 2019; Mide et al., 2023; Orgaz et al., 2013; Sancho et al., 2021; Schluter et al., 2019; Shirk et al., 2018) and community samples (Labrador et al., 2020; Matsushita et al., 2025; Nigro et al., 2021; Thurm et al., 2022). However, some studies have failed to find significant associations between cognitive distortions and gambling severity (Mallorquí-Bagué et al., 2019; Mide et al., 2023; Yokomitsu et al., 2021), suggesting that this association may be influenced by other individual, gambling-related, or contextual factors.

Based on the I-PACE model (Brand et al., 2016), cognitive distortions are expected to mediate the relationship between gambling modality and type, and gambling severity because the structural and situational characteristics of different gambling formats shape gamblers’ cognitive processing and foster specific erroneous beliefs about chance, skill, and control. These cognitive distortions, in turn, influence subsequent decisions and emotional reactions during gambling (e.g., encouraging continued gambling, justifying losses, and promoting an overestimation of personal skill) and may therefore provide the mechanism through which certain gambling modalities lead to more severe gambling problems.

Given the well-documented gender differences in gambling behavior, such as variations in severity (Secades-Villa et al., 2024), types of games (Mccormack et al., 2014; Palmer Du Preez et al., 2021), gambling motives (Merkouris et al., 2016), and associated risk factors (Hing et al., 2016; Macía et al., 2025), it is reasonable to expect that gender also influences the presence and type of cognitive distortions. Previous evidence highlights that gender may play a significant role in the manifestation and impact of gambling-related cognitions, although empirical research on this topic remains limited. Overall, studies have shown that males tend to exhibit higher levels of cognitive distortions than females across most dimensions, particularly regarding erroneous beliefs about control and prediction. However, no consistent gender differences have been observed in the “illusion of control” dimension, where findings are generally nonsignificant (Ciccarelli et al., 2021; Cosenza et al., 2019; Estévez et al., 2021). Other studies suggest a more nuanced pattern, indicating that while females tend to report higher gambling-related expectancies, males score higher on illusion of control, predictive control, interpretative bias, and the total score for cognitive distortions, with no gender differences observed in the inability to stop (Estévez et al., 2021).

Despite robust evidence supporting the association between cognitive distortions and gambling behavior, prior research presents several noteworthy limitations. A substantial proportion of studies have focused on adult populations (Mallorquí-Bagué et al., 2019; Mide et al., 2023; Nigro et al., 2021) or university students (Esparza-Reig et al., 2022, 2023; Ubillos-Landa et al., 2022; Yokomitsu et al., 2021), leaving a significant gap in understanding the underlying mechanisms during adolescence, a critical developmental period for prevention efforts. Additionally, most studies were conducted with clinical samples composed of individuals already seeking treatment (Koga et al., 2025; Mallorquí-Bagué et al., 2019; Mide et al., 2023; Sancho et al., 2021; Schluter et al., 2019; Shirk et al., 2018) or even combined clinical and community samples (Bonnaire et al., 2022; Ciccarelli et al., 2017; Estévez et al., 2021). Moreover, the predominance of male participants in most samples (e.g., over 85%) (Koga et al., 2025; Matsushita et al., 2025; Orlowski et al., 2020) has hindered robust comparative analyses of cognitive distortions and gambling severity among females, despite evidence suggesting that gender may play a pivotal role in these distortions (Ciccarelli et al., 2021; Cosenza et al., 2019; Estévez et al., 2021). Furthermore, only a limited number of studies have examined differences based on the gambling type (i.e., strategic vs. non-strategic games) (Lévesque et al., 2018; Mallorquí-Bagué et al., 2019; Myrseth et al., 2010; Orlowski et al., 2020), or the gambling modality (i.e., online vs. offline) (Mallorquí-Bagué et al., 2019; Sancho et al., 2021). Given the variability across gambling activities and modalities in other relevant domains (e.g., severity, risk factors) (Secades-Villa et al., 2024; Jiménez-Murcia et al., 2020; Leslie & McGrath, 2023), it is important to investigate whether cognitive distortions differ accordingly. Notably, to our knowledge, no study has examined whether cognitive distortions mediate the relationship between gambling type or modality and gambling severity.

Against this background, the present study aims to examine gambling-related cognitive distortions by analyzing differences according to gambling severity (i.e., non-problematic, at-risk, and problematic gambling), gambling modality (i.e., online vs. land-based/offline), and gambling type (i.e., strategic vs. non-strategic gambling). In addition, the study will assess the mediating role of cognitive distortions in the relationship between gambling modality, gambling type, and gambling severity. To provide a more nuanced understanding of these patterns, all analyses were conducted separately for men and women, enabling the identification of gender-related differences in the manifestation of cognitive distortions across gambling profiles.

## Methods

### Participants

The study included 209 adolescents who reported past-year gambling and were enrolled in secondary education, ranging in age from 14 to 18 years (*M* = 15.72, *SD* = 1.05). The sample was composed of 30.9% girls (*n* = 63), and 69.1% boys (*n* = 141). The remaining 5 participants did not complete this variable. Participants were distributed across educational levels, with 20.1% in the third year of compulsory secondary education (9th grade in the US), 27.8% in the fourth year (10th grade in the US), 2.9% enrolled in vocational training programs, 38.8% in the first year of baccalaureate (11th grade in the US), and 10.0% in the second year of baccalaureate (12th grade in the US). Regarding nationality, only 39.2% (*n* = 82) of the participants provided this information. Among those who responded, the majority were of Spanish nationality (92.7%, *n* = 76), while 7.3% (*n* = 6) reported a different nationality.

To justify the adequacy of this sample for the structural equation model, an a priori power analysis was conducted based on the RMSEA model fit criterion (Moshagen & Bader, 2023). With 598 degrees of freedom and an alpha level of 0.05, the analysis revealed that our sample size provides a statistical power exceeding 0.99 to detect differences from a good fit (RMSEA = 0.05), confirming that the study is sufficiently powered.

### Measures

#### Gambling Behavior

Gambling behavior was assessed in terms of both lifetime and past-year prevalence. In addition, participants were asked to indicate the types of gambling they had engaged in the following gambling activities: (1) card games involving money (e.g., poker), (2) betting on horse races or rural sports, (3) participation in lotteries, (4) casino games, (5) bingo, (6) slot machines, and (7) sports betting. Additionally, they had to specify the modality, that is, online, offline/in-person, or both.

Following previous research (Jiménez-Murcia et al., 2020; Moragas et al., 2015), participants were classified into three groups according to the type of gambling activity they engaged in: strategic gamblers, who participated exclusively in games emphasizing individual skill and decision-making (e.g., sports betting, card games); non-strategic gamblers, who gambled only games of chance (e.g., electronic gambling machines, slots, lotteries, bingo); and mixed gamblers, who engaged in both strategic and non-strategic gambling activities.

To estimate the severity of gambling involvement, the study employed the South Oaks Gambling Screen-Revised for Adolescents (SOGS-RA) (Winters et al., 1993), an adaptation of the original adult version developed by Lesieur and Blume (1987). The Spanish validation for adolescents was used in the present study (Becoña, 1997; Becoña et al., 2001). The SOGS-RA includes 12 dichotomous items, yielding a total score ranging from 0 to 12, where higher scores indicate greater gambling severity. Based on established cutoffs, participants were classified as non-gamblers/non-problem gamblers (0–1), at-risk gamblers (2–3), or problem gamblers (≥ 4). In this sample, the instrument demonstrated excellent internal consistency (Cronbach’s α = 0.867).

#### Gambling-Related Cognitive Distortions

Cognitive distortions related to gambling were assessed using the Gamblers’ Beliefs Questionnaire (GBQ) (Steenbergh et al., 2002), in its Spanish adaptation and validation by Ubillos-Landa et al. (2022). The GBQ is a self-report instrument composed of 21 items designed to measure irrational beliefs and misperceptions associated with gambling behavior. Each item is rated on a 7-point Likert scale ranging from 1 (strongly disagree) to 7 (strongly agree). Higher scores indicate a greater endorsement of gambling-related cognitive distortions. Although the instrument was originally developed under a two-factor model (i.e., ‘Illusion of Control’ and ‘Belief in Luck and Perseverance’) the present study utilized the GBQ total score. This decision was empirically supported by the extremely high correlation between the subscales and the subsequent detection of significant multicollinearity, suggesting that a composite measure provides a more stable and parsimonious representation of cognitive distortions in this sample. In the present study, the internal consistency for the total scale was excellent (α = 0.979).

### Procedure

Participants were recruited from a range of educational institutions across Spain, including both secondary schools and vocational training centers. All schools received detailed information about the study and its objectives prior to participation. Before starting, students were fully informed about the study’s purpose and procedures. Written informed consent was obtained, confirming that participation was voluntary and that students understood the process. Participation was optional, no incentives were offered, and students were assured that non-participation would have no academic or personal consequences. All responses were collected anonymously and treated with strict confidentiality. Data collection was conducted during regular class periods, with students completing the questionnaire under the supervision of either a researcher or their teacher. Completion time averaged approximately 25 min. Data were collected between February and March 2025. The study protocol was approved by the Ethics Committee of the University of Deusto [(ref.: ETK-59/23-24 [) and conducted in accordance with the ethical guidelines of the Declaration of Helsinki.

### Data Analysis

First, the internal consistency of the SOGS-RA and the GBQ was assessed using Cronbach’s alpha (Cronbach, 1951), with an optimal cutoff point set at 0.80 (Doval et al., 2023). Descriptive statistics were calculated for quantitative variables, and frequencies were computed for categorical variables.

To examine differences in cognitive distortions, analysis of variance (ANOVA) was conducted according to gambling severity (non-problem, at-risk, and problematic), gambling modality (online, offline, or both), and type of game (strategic, non-strategic, or both). Separate ANOVAs were performed for the total sample and stratified by gender (male and female samples). When the initial omnibus tests were statistically significant, post-hoc pairwise comparisons were conducted according to the distributional and variance properties of the data. Because the dependent variable (i.e., GBQ total score) followed a normal distribution and met the assumption of homoscedasticity, Bonferroni post hoc tests were applied (Juarros-Basterretxea et al., 2024). Effect sizes were estimated using omega squared (ω²), interpreted as follows: values below 0.01 were considered very small, values between 0.01 and 0.06 were considered small, values between 0.06 and 0.14 were considered medium, and values above 0.14 were considered large. To control the family-wise Type I error rate associated with conducting multiple ANOVAs, *p*-values were adjusted using the Holm-Bonferroni sequential correction procedure (Holm, 1979). For each family of tests, raw *p*-values were ordered from smallest to largest and sequentially compared against adjusted significance thresholds α/(m-i + 1), where *m* represents the total number of comparisons and *i* the rank of the ordered *p*-value. This method provides a uniformly more powerful alternative to the classical Bonferroni correction while maintaining strong control over the family-wise error rate.

To examine the mediating role of cognitive distortions, a Multi-Group Structural Equation Model (MG-SEM) was performed, with gender as the grouping variable. The model was specified by including four dichotomous independent variables (yes/no) representing gambling modalities (i.e., offline gambling and online gambling) and type of gambling (i.e., strategic gambling and non-strategic gambling). Cognitive distortions were employed as the mediating variable, treated as a single latent factor (comprising all 21 items of the GBQ); this decision was made to ensure model parsimony and stability following the detection of significant multicollinearity among the original subscales (tolerance ≤ 0.165; VIF ≥ 6.049). The dependent variable was gambling severity, as measured by the SOGS total score. Missing data were handled using the Full Information Maximum Likelihood (FIML) method.

Model fit was estimated using maximum likelihood estimation with a multipronged approach. While a chi-square test was performed, its known sensitivity to sample size led us to prioritize alternative fit indices: the Tucker-Lewis index (TLI) and the Comparative Fit Index (CFI), with values > 0.90 indicating adequate fit, and the root mean square error of approximation (RMSEA), with values < 0.08 suggesting acceptable fit (Byrne, 2009; Hu & Bentler, 1999; Schermelleh-Engel et al., 2003).

Furthermore, measurement invariance was tested to ensure construct comparability across genders. Metric invariance was established, as the decrease in the CFI was less than 0.010 and the increase in the RMSEA was less than 0.015 compared to the configural model (Chen, 2007). Once invariance was confirmed, indirect effects were estimated using a 5,000-sample bootstrap procedure with 95% bias-corrected confidence intervals, ensuring that mediation findings reflected stable and robust structural relationships.

Outliers were assessed using boxplots and standardized z-scores, with all scores were below |3.29|, indicating no extreme cases. All analyses were conducting using JASP (version 0.18.3.0), applying a 95% confidence interval.

## Results

### Cognitive Distortions Based on Gambling-Related Variables

Statistically significant differences with a medium effect size were found in the total score of cognitive distortions across levels of gambling severity (*p* = .003, ω^2^ = 0.104). Post hoc comparisons revealed that cognitive distortions increased with gambling severity, with individuals showing problematic gambling reporting significantly higher scores than those with non-problematic gambling (97.94 vs. 67.61).

For gambling modality, statistically significant differences with a medium effect size were observed across online, offline, and mixed-modality gamblers (*p* = .003, ω^2^ = 0.086). Exclusively online gamblers exhibited significantly higher levels of total distortions compared with exclusively offline gamblers (94.86 vs. 68.00). Regarding the type of gambling (i.e., strategic vs. non-strategic), small but significant differences were found (*p* = .024, ω^2^ = 0.038). While the highest scores were observed in participants involved in both strategic and non-strategic activities (*M* = 79.88, *SD* = 31.34), post-hoc analysis did not reveal significant pairwise differences between specific groups (see Table 1).

**Table 1:** Cognitive distortions across gambling severity, gambling modality, and type of gambling, in the total sample and by gender

Variables	Total sample (*N* = 209)	Male (*n* = 141)	Female (*n* = 63)
Gambling severity			
Non-problematic gambling	67.61 (35.07)^a^	68.75 (33.05)^a^	65.90 (37.01)^a^
At-risk gambling	81.22 (31.09)^a.b^	79.23 (31.09)^a.b^	91.50 (31.75)^a^
Problematic gambling	97.94 (25.72)^b^	97.50 (24.61)^b^	96.00 (33.45)^a^
*p*-value (ω²)	0.003 (0.104)	0.003 (0.105)	0.132 (0.058)
Gambling modality			
Online	94.86 (33.43)^a^	85.20 (25.22)^a^	114.60 (44.65)^a^
Offline	68.00 (34.71)^b^	73.46 (34.79)^a^	61.19 (33.42)^b^
Mixed	86.92 (29.39)^a.b^	85.05 (29.20)^a^	90.50 (31.36)^a.b^
*p*-value (ω²)	0.003 (0.086)	0.154 (0.016)	0.009 (0.192)
Type of gambling			
Strategic	63.64 (27.98)^a^	66.36 (28.09)^a^	43.67 (20.82)^a^
Non-strategic	66.17 (31.70)^a^	60.88 (32.09)^a^	68.81 (32.22)^a^
Both	79.88 (31.34)^a^	80.71 (29.44)^a^	70.37 (34.63)^a^
*p*-value (ω²)	0.024 (0.038)	0.092 (0.041)	0.428 (0.000)

Note. All *p*-values were adjusted using the Holm–Bonferroni correction for multiple comparisons. Different superscript letters indicate statistical significance at a 95% confidence interval. In a given row, entries with superscript (a) have significantly different means from entries with superscript (b)

### Gambling-Related Distortions as a Function of Gender

Among men, significant differences were found in total cognitive distortions across gambling severity levels (*p* = .003, ω^2^ = 0.105), with higher distortions associated with increasing severity. Specifically, those with problematic gambling reported significantly higher levels of distortions than those with non-problematic gambling (97.50 vs. 68.75). In contrast, no significant differences emerged across gambling modalities (*p* = .154, ω^2^ = 0.016) or type of gambling (*p* = .092, ω^2^ = 0.041) for the male subsample.

Among female participants, no significant differences were found in cognitive distortions based on gambling severity (*p* = .132, ω^2^ = 0.058). However, significant differences were observed across gambling modalities (*p* = .009, ω^2^ = 0.192), showing a large effect size. Specifically, female online gamblers reported markedly higher cognitive distortions compared with exclusively offline female gamblers (114.60 vs. 61.19). Finally, the type of gambling did not yield significant differences (*p* = .428, ω^2^ = 0.000) among women (see Table 1).

### The Mediating Role of Gambling-Related Cognitive Distortions in the Relationship Between Gambling Modality and Type and Gambling Severity

**Fig. 1:**
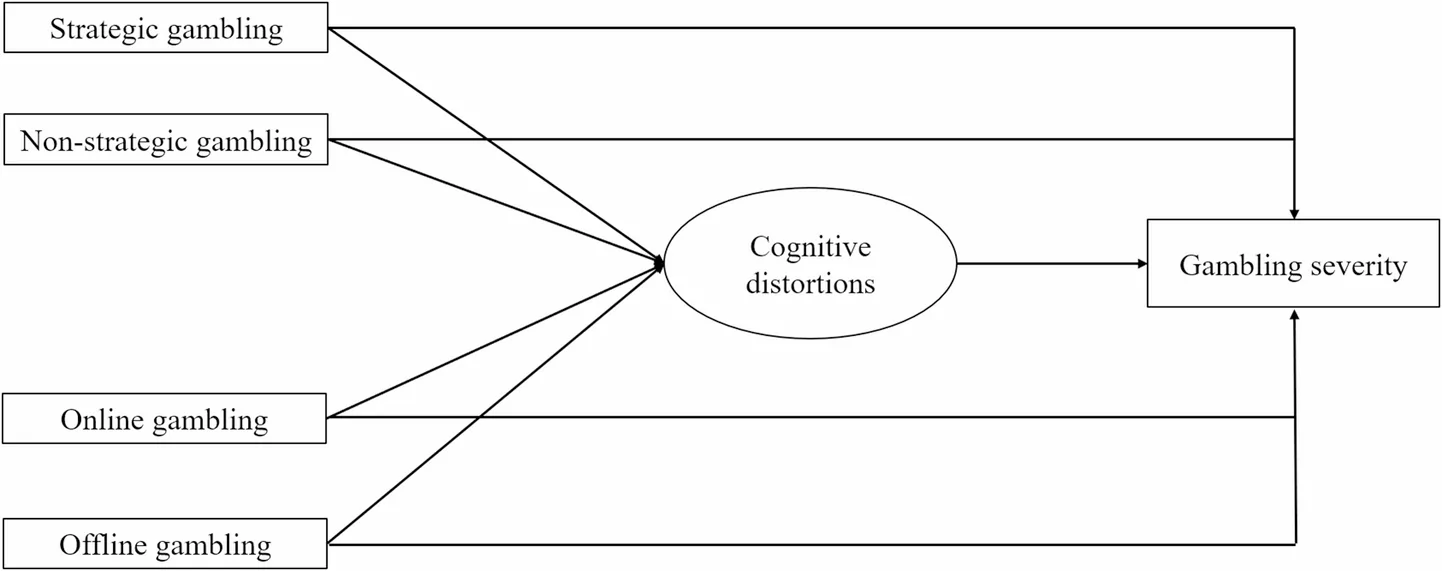
Mediation model linking gambling type and modality, cognitive distortions, and gambling severity

To examine the mediating role of gambling-related cognitive distortions in the relationship between gambling modality, gambling type, and gambling severity, a MG-SEM was conducted. Prior to evaluating the structural paths, measurement invariance across gender was assessed to ensure the comparability of the model. The diagram of the mediation model is presented in Fig. 1. All direct, indirect, and total effects (both standardized and unstandardized), including confidence intervals, are reported in Table 2.

**Table 2:** Direct, indirect, and total effects of gambling type and gambling modality on gambling severity

	Female					Male				
	B	β	*p*-value	LLCI	ULCI	B	β	*p*-value	LLCI	ULCI
Direct effects										
Strategic gambling ◊ Gambling severity	0.480	0.480	0.386	− 0.605	1.565	0.213	0.213	0.710	− 0.909	1.335
Non-strategic gambling ◊ Gambling severity	− 0.086	− 0.086	0.869	-1.116	0.943	0.771	0.771	0.117	− 0.193	1.736
Online gambling ◊ Gambling severity	− 0.040	− 0.040	0.948	-1.242	1.161	1.573	1.573	< 0.001	0.652	2.495
Offline gambling ◊ Gambling severity	0.217	0.217	0.689	− 0.846	1.281	− 0.387	− 0.387	0.434	-1.355	0.581
Cognitive distortions ◊ Gambling severity	0.387	0.699	0.006	0.111	0.664	0.495	0.788	< 0.001	0.226	0.763
Strategic gambling ◊ Cognitive distortions	− 0.444	− 0.246	0.380	-1.437	0.548	− 0.296	− 0.186	0.420	-1.016	0.424
Non-strategic gambling ◊ Cognitive distortions	0.413	0.229	0.391	− 0.529	1.354	0.195	0.122	0.537	− 0.424	0.814
Online gambling ◊ Cognitive distortions	1.682	0.932	0.001	0.644	2.720	0.806	0.506	0.007	0.224	1.389
Offline gambling ◊ Cognitive distortions	− 0.434	− 0.240	0.382	-1.406	0.539	0.361	0.226	0.254	− 0.259	0.981
Indirect effects										
Strategic gambling ◊ Cognitive distortions ◊ Gambling severity	− 0.172	− 0.172	0.402	− 0.965	0.050	− 0.146	− 0.146	0.431	− 0.563	0.224
Non-strategic gambling ◊ Cognitive distortions ◊ Gambling severity	0.160	0.160	0.411	− 0.085	1.197	0.096	0.096	0.543	− 0.162	0.433
Online gambling ◊ Cognitive distortions ◊ Gambling severity	0.651	0.651	0.035	0.076	2.141	0.399	0.399	0.028	0.116	0.888
Offline gambling ◊ Cognitive distortions ◊ Gambling severity	− 0.168	− 0.168	0.404	-1.188	0.116	0.178	0.178	0.275	− 0.102	0.592
Total effects										
Strategic gambling ◊ Gambling severity	0.308	0.308	0.598	− 0.835	1.450	0.066	0.066	0.911	-1.106	1.239
Non-strategic gambling ◊ Gambling severity	0.073	0.073	0.895	-1.011	1.157	0.868	0.868	0.092	− 0.141	1.876
Online gambling ◊ Gambling severity	0.611	0.611	0.308	− 0.565	1.788	1.972	1.972	< 0.001	1.034	2.911
Offline gambling ◊ Gambling severity	0.049	0.049	0.931	-1.070	1.169	− 0.208	− 0.208	0.686	-1.217	0.801

Note. LLCI = lower-limit confidence intervals; ULCI = upper-limit confidence interval

The evaluation of invariance was based on the comparison of the configural model (unconstrained) and the metric model (where factor loadings were constrained to be equal across groups). The results supported metric invariance, as the change in fit indices remained within recommended thresholds (ΔCFI = 0.001; ΔRMSEA = − 0.003). The final MG-SEM showed the following fit indices: χ^2^ = 1,411.70, *p* < .001, CFI = 0.926, TLI = 0.912, RMSEA = 0.066 (90%CI [0.029, 0.104]), and SRMR = 0.051.

The structural model revealed significant associations for both groups. Among male participants, gambling severity was directly associated with both online gambling modality (β = 1.573, *p* < .001) and cognitive distortions (β = 0.788, *p* < .001). Furthermore, online gambling was positively linked to cognitive distortions (β = 0.506, *p* = .007). A significant indirect association was observed between online gambling and gambling severity through the mediating role of cognitive distortions (β = 0.399, *p* = .028).

For female participants, a significant direct relationship was found between cognitive distortions and gambling severity (β = 0.699, *p* = .006), and between online gambling and cognitive distortions (β = 0.932, *p* = .011). Most notably, the model identified a significant indirect association between online gambling and gambling severity, mediated by cognitive distortions (β = 0.651, *p* = .035).

## Discussion

This descriptive study aimed to examine the relationship between cognitive distortions and gambling severity (i.e., non-problematic, at-risk, and problematic gambling), gambling modality (i.e., online vs. land-based/offline), and gambling type (i.e., strategic vs. non-strategic gambling) in the adolescent population. In addition, the mediating role of cognitive distortions in the relationship between gambling type and modality, and gambling severity was tested. Notably, these associations were explored separately for males and females to better understand potential gender-specific patterns.

Overall, higher levels of cognitive distortions were observed among adolescents reporting greater gambling severity and among those who engaged in online gambling. These findings are consistent with the cognitive model of gambling behavior, which proposes that cognitive distortions are associated with greater involvement in gambling activities and are closely linked to problematic engagement. Previous research has repeatedly documented associations between gambling severity and cognitive distortions across both adolescent (Calado et al., 2017; Ciccarelli et al., 2021; Cosenza et al., 2019) and adult populations (Bonnaire et al., 2022; Ciccarelli et al., 2017; Labrador et al., 2020; Nigro et al., 2021). Current results extend this pattern to a community sample of adolescents who reported past-year gambling involvement.

Given the importance of understanding gender-specific pathways in gambling behavior (McCarthy et al., 2019), when analyses were stratified by gender, a differentiated pattern emerged. Among male adolescents, cognitive distortions increased progressively across levels of gambling severity, with individuals exhibiting problematic gambling reporting markedly higher scores than those with non-problematic gambling. This pattern aligns with prior literature indicating a stronger endorsement of erroneous gambling-related beliefs among males and suggests that, in boys, cognitive distortions tend to co-occur with the escalation of gambling involvement (Ciccarelli et al., 2021; Cosenza et al., 2019; Estévez et al., 2021). In contrast, among female adolescents, cognitive distortions did not significantly vary by gambling severity but were higher among those who engaged in online gambling compared to offline gambling. Importantly, this outcome may reflect that the cognitive distortions assessed in the present study do not fully capture activity-specific or situationally activated cognitions that could be more closely linked to the gambling activities more frequently preferred by female adolescents. Prior research has suggested that gambling-related cognitions are not necessarily stable deficits but may be motivationally driven and dependent on game characteristics and contextual activation (Mallorquí-Bagué et al., 2019; Muela et al., 2020; Perales et al., 2017).

This gender-specific pattern highlights the relevance of the online environment for the development of cognitive distortions in girls. While some adult studies have reported higher distortions among online gamblers (Mallorquí-Bagué et al., 2019), others have not identified modality-related differences (Sancho et al., 2021). Online gambling provides rapid, continuous play with immediate feedback and visually salient wins, which may increase the likelihood of interpreting outcomes as predictable or controllable, reinforcing cognitive distortions (Auer & Griffiths, 2023; Torrance et al., 2024).

Regarding gambling type, only small differences were observed, and post-hoc comparisons were not significant, indicating that the strategic versus non-strategic classification was not strongly associated with cognitive distortions in this sample. This contrasts with theoretical assumptions suggesting that strategic games should be more strongly related to illusion-of-control beliefs (Mallorquí-Bagué et al., 2019; Myrseth et al., 2010). During adolescence, the perception of skill may not yet be clearly differentiated across gambling activities, and both strategic and non-strategic games may be interpreted through similar cognitive beliefs. Consequently, structural characteristics of the gambling environment, particularly online accessibility, appear more relevant than the formal skill component when considering cognitive distortions in this population.

Beyond the descriptive differences, the MG-SEM allowed for the examination of the associations between gambling modality, type of gambling, cognitive distortions, and gambling severity within a single framework. Across both genders, cognitive distortions were positively associated with gambling severity. This finding is consistent with theoretical models proposing that gambling-related cognitions are closely intertwined with maladaptive engagement in gambling activities (see e.g., Bonnaire et al., 2022; Cosenza et al., 2019; Mancone et al., 2025; Orlowski et al., 2020; Thurm et al., 2022), particularly in developmental stages characterized by the ongoing maturation of decision-making and probabilistic reasoning (Edelson & Reyna, 2023; Primi et al., 2016).

However, the pattern of associations linking online gambling and severity differed between males and females. In males, online gambling was associated with gambling severity both directly and indirectly through cognitive distortions. This suggests that while cognitive distortions are associated with severity, other factors may also contribute to gambling problems (e.g., gambling motives, psychological-related variables) (Aonso-Diego et al., 2024; Jiménez-Murcia et al., 2019; Leslie & McGrath, 2023). In females, online gambling was related to higher levels of cognitive distortions, and these, in turn, were associated with greater gambling severity, whereas the direct association between online gambling and severity was not significant. Taken together, these gender-specific patterns are in line with integrative frameworks such as the I-PACE model, which proposes that the interaction between individual characteristics and situational features of digital environments shapes addictive behaviors (Brand et al., 2016). These findings highlight the relevance of the online gambling environment in the study of adolescent gambling-related cognitions. Across genders, online participation was associated with higher cognitive distortions regardless of gambling type (strategic vs. non-strategic), suggesting that structural features of digital platforms (e.g., rapid feedback, continuous play, and salient near-misses) may be closely linked to biased interpretations of outcomes.

### Limitations

This study has several limitations that should be considered when interpreting the results. First, its cross-sectional design prevents any inference of causality between cognitive distortions and gambling behavior. Second, although cognitive distortions were assessed with the GBQ, the use of a more comprehensive instrument, such as the Gambling-Related Cognition Scale (GRCS) could have provided a broader assessment of gambling-related cognitive biases. Third, the GBQ primarily measures stable and global cognitive distortions (see e.g., Mallorquí-Bagué et al., 2019; Muela et al., 2020; Perales et al., 2017), whereas gambling-related cognitions can also be state-dependent and triggered by the gambling situation itself. This may have limited the detection of situational or game-specific distortions, particularly in female adolescents who preferred different gambling modalities (Mccormack et al., 2014; Palmer Du Preez et al., 2021). Fourth, some gambling activities, such as roulette, were not included in the assessment, and participation in emerging online contexts that blend gaming and gambling (e.g., loot boxes, in-game microtransactions, or online trading) was also not evaluated. Given the growing prevalence of these behaviors among adolescents, future research should consider incorporating them to better capture the evolving spectrum of gambling-like activities (Coloma-Carmona et al., 2025; Delfabbro et al., 2021; Drummond et al., 2020; National Plan on Drugs, 2025). Additionally, the casino category was excluded from the strategic/non-strategic classification due to its ambiguity. Fifth, the comparisons of cognitive distortions across gambling profiles were conducted using ANOVA without adjusting for potential confounders, including sociodemographic (e.g., age, socioeconomic status), psychological (e.g., impulsivity, emotion regulation), social (e.g., parental monitoring, school environment), or contextual variables (e.g., exposure to marketing). The absence of these covariates may have influenced the observed group differences and limits the interpretability of the results. Future research should employ multivariate designs to account for these factors.

## Conclusion

The findings of the present study emphasize the strong association between gambling-related cognitive distortions and gambling behavior in the adolescent population, showing that this association is characterized by different patterns of association for boys and girls, and offering a more nuanced understanding of how certain gambling patterns are differentially associated with greater problematic behavior during this developmental stage. Notably, these results highlight the need to examine cognitive distortions separately in adolescent boys and girls, as the pattern of association between online gambling and severity differs by gender rather than merely their magnitude, and gender differences may manifest differently during adolescence than in adulthood.

As has been noted in previous research, gambling studies typically include disproportionately high numbers of men, which can obscure distinct patterns that emerge when gender is considered. Our findings illustrate that such masking effects may be particularly relevant when analyzing the role of cognitive distortions in different gambling modalities, particularly within online gambling. These findings present meaningful clinical implications. Prior evidence indicates that addressing cognitive distortions is associated with improvements in gambling behavior (Eriksen et al., 2023; Martín-Pérez et al., 2025; Williams et al., 2010, 2012), and distortions have been identified as significant predictors of relapse and treatment dropout among individuals with gambling problems (Mallorquí-Bagué et al., 2019). In this context, the present results underscore the importance of incorporating gender-sensitive considerations into prevention and intervention strategies targeted at adolescents. For boys, online gambling was directly and indirectly associated with gambling severity through cognitive distortions, suggesting that interventions may need to consider multiple correlated risk factors beyond cognitive distortions alone. For girls, online gambling showed an indirect statistical association with gambling severity via cognitive distortions, indicating that interventions may particularly benefit from targeting beliefs related to digital gambling environments. More broadly, this perspective reinforces the idea that the interplay between cognitive distortions, gambling type, and individual characteristics (e.g., gender) may help clarify why certain gambling behaviors are more closely linked to problematic patterns in some groups than in others. Taken together, these insights support the need for gender-based approaches in both the clinical assessment and treatment of gambling-related problems that consider distinct developmental patterns of association rather than only prevalence differences.

Future research should incorporate real-time assessment of gambling-related cognitive distortions using ecological momentary assessment methodologies to better capture proximal risk processes (Dowling et al., 2021; Hawker et al., 2026). It will also be important to include emerging gambling-like activities (e.g., loot boxes in videogames, online trading, cryptocurrencies) given their increasing presence in adolescents’ daily lives (National Plan on Drugs, 2025). In addition, further work should continue examining the role of gender across diverse populations and developmental stages. Longitudinal designs and multimethod approaches (e.g., behavioral and digital trace data) would also help clarify causal mechanisms and improve the ecological validity of findings, ultimately informing more tailored prevention and intervention strategies. 

## Data Availability

The data that support the findings of this study are available from the corresponding author upon reasonable request.
